# Occupational safety practice among metal workers in Bangladesh: a community-level study

**DOI:** 10.1186/s12995-022-00366-y

**Published:** 2022-12-12

**Authors:** Farhin Islam, MR Alam, SM Abdullah AL Mamun, Mohammad Sorowar Hossain

**Affiliations:** 1 Department of Emerging and Neglected Diseases, Biomedical Research Foundation, House- 22, 6th Floor, Road- 08, Sector- 09, Uttara, Dhaka, 1230 Bangladesh; 2 Department of Environmental Science and Management, Independent University, Bangladesh (IUB), Dhaka, Bangladesh

**Keywords:** Occupational health and safety, Workplace injury, OHS knowledge, OHS practices, Metal works, Regulation

## Abstract

**Background:**

The overall information on occupational health and safety (OHS)-related knowledge and workplace practices are scarce in Bangladesh. This study aimed to (i) examine the prevalence of occupational injuries, (ii) explore the level of OHS-related knowledge and practice among workers and associated factors, and (iii) investigate the socioeconomic factors and OHS-related knowledge and practice scores as determinants of injury among metal workers at a community setting in Bangladesh.

**Methods:**

This was a cross-sectional study conducted on all the functional metal workshops in a community of a town. The sociodemographic characteristics, history of injury and its consequences, and the state of knowledge and practice were measured using descriptive statistics. Univariate and multivariate analyses were used to measure the association between practice scores and sociodemographic factors and knowledge. Logistic regression was conducted to get the odds ratio of getting injured.

**Results:**

A high annual rate (82.9%) of occupational injuries was documented in a one-year timeframe and the majority (81.1%) of injured workers lost more than three working days (median 20 days). Workers working in workshops with more than three workers were 3.3 times more likely to be injured [AOR = 3.33, 95% CI = 1.16, 9.58] compared to the workers in factories with one to three workers. Most of the workers had the basic knowledge related to OHS but the mean practice score was very low, 1.86 (SD 1.17). Higher education, lower monthly family income, and being an owner significantly led to higher practice scores.

**Conclusions:**

The OHS-related knowledge was not properly translated into good workplace practices in small informal metal workshops because of the absence of implementation of OHS policies and monitoring by the relevant authority. Government should support the informal metal working sector to increase awareness and skills for the prevention and proper management of injuries and risks, and to ensure access to safety equipment and a safe environment.

## Background

Occupational injuries are considered a major public health concern worldwide, particularly in low and middle-income countries. Work-related hazards claim nearly 2.78 million lives each year globally and around 2.4 million of those fatalities are caused by occupational diseases [1]. Additionally, medical treatment for workplace accidents or inability to work, which can cause more than 4 days of absence, affects 374 million employees every year. The cost associated with lost working days and health care expenditure is projected to account for about a 4% loss of global GDP [2]. Hence, workers’ protection from sickness, disease, and injury as a consequence of their occupational risk is the core component of the constitution of the International Labour Organization (ILO) [3].

Bangladesh is one of the densely populated lower-middle-income countries with a population of around 165 million [4]. In 2017, the National Labor Force Survey (LFS) estimated that 1.9 million persons (3.1% of the total employed) aged 15 or older experienced at least an occupational injury within a year [5]. However, the overall information on occupational health and safety (OHS)-related knowledge and workplace practices are scarce in Bangladesh.

Following the devastating collapse of a garment factory (named Rana Plaza) in 2013 that caused 1132 deaths [6], Bangladesh took initiative to develop OHS-related policies. Consequently, Bangladesh Labour Act (Amendment), 2013, Bangladesh Labour Rule 2015, and National Occupational Health and Safety (OHS) Policy 2013 include mandatory use of personal safety equipment, the formation of health centres in large companies, the establishment of the safety committee, maintaining standard OHS protection, and creating a strategy and action plan [7]. But the National OHS Policy 2013 could not achieve its goal because there are challenges in implementing this policy in workplaces, which may include a lack of coordination among stakeholders, the absence of coordinating authority, the lack of monitoring and accountability, and a lack of awareness among firms and workers.

In Bangladesh, the metal products manufacturing industry represents 0.4% of total employment [8]. The market size of the steel industry alone is around USD 5.3 billion [9]. Around 3,76,000 males and 33,000 females are employed in the manufacturing of basic metals or fabricated metal products: 2,14,000 in urban areas and 1,95,000 in rural areas [10]. Most of the metal workshops are small and medium-sized enterprises (SMEs) with less than 20 employees.

Even though most workplace fatalities and injuries are preventable through generating awareness and implementation of OHS guidelines and legislation, few studies on OHS in the metalworks industry have been conducted in Bangladesh [11, 12]. None of these studies addressed the knowledge and practice of OHS among metal workers and the sociodemographic factors associated with injuries. Hence, this study aimed to (i) examine the prevalence of occupational injuries, (ii) explore the state of OHS-related knowledge and practice among workers and associated factors, and (iii) investigate the socioeconomic factors and OHS-related knowledge and practice scores as determinants of injury among metal workers at a community setting in Bangladesh.

## Materials and method

### Study setting and participants

This cross-sectional study was conducted in Jamalpur Pauroshova (also known as town) which is the administrative centre of the Jamalpur district. The study area (Jamalpur pauroshova or town) consists of urban and peri-urban areas with a population of nearly 2,00,000. The demographic and socioeconomic factors of the study area are similar to the other 33 district towns (out of 64) of Bangladesh [13]. In general, large metal industries are rarely available at the district level, rather small metal workshops are available to support local needs.

### Sampling

As there is a lack of a systematic registration system for metal workshops at the community level and so we listed all functional metal workshops for our study purpose. In this study, we approached one person in each workshop (preferably the owner or a senior employee) of all (*n* = 258) listed metal workshops for data collection. The response rate was 80.4% (*n* = 205).

### Questionnaire and data collection

Data was collected through a structured questionnaire. The research team prepared the questionnaire based on a careful understanding of similar previous studies from India and Ethiopia [14–16]. The questionnaire has 31 questions in total to address the identification of the workshop, demographic characteristics, history of injury, practice in the workplace, and knowledge about occupational safety. Firstly, demographic data has been collected on the aspects of age, education, occupation, monthly income, marital status, type of metalwork, number of workers, age of the firm, self-reported OHS status of the workplace, and perception towards PPE and safety training. Secondly, the history of injury includes information on the frequency of injury in the last year, type of injury, immediate response, and the number of lost working days. Thirdly, the workers’ safety knowledge was tested using four questions about the health impacts of dust, noise, spark, and protective measures and equipment. To keep the questionnaire concise, only four essential knowledge-related questions were kept in the questionnaire since the main focus of the study was on OHS practices. Fourthly, the practices of the workers were assessed using questions related to protective measures, first aid, rules and protocols, awareness programs and discussions, training programs, and manual work. All questions regarding knowledge and practice were close-ended and categorical. The questionnaire was piloted among five workers, and then it was revised and finalized. Finally, the study participants were interviewed from 21 January 2020 to 22 February 2020 by nine enumerators. All enumerators were trained beforehand for face-to-face interviews to collect data.

### Knowledge and practice scoring

Each “Yes” (satisfactory) answer in the practice section carries 1 mark while each “No” (unsatisfactory) or “Don’t know” (uncertain) answer carries 0 marks. Only one question in the practice section, which says whether manual handling is very common or not in operation, carries different markings (1 mark for “No” and 0 for “Yes”). As there are nine items in the practice section, the total practice score is ranged between 0 and 9. This scoring system is adapted from Khan et al. [17]. The knowledge score is calculated following the same pattern but the score has a range from 0 to 4 as there are four items in the knowledge section.

### Data management and statistical analysis

The collected data were entered into REDCap (Research Electronic Data Capture) System. Data was exported from REDCap to the statistical package Stata version 13 for analysis. The sociodemographic characteristics, history of injury and its consequences, and the state of knowledge and practice were measured using descriptive statistics. Univariate and multivariate analyses have been used to measure the association between practice scores and sociodemographic characteristics, knowledge, and other variables; Logistic regression provided the odds ratio of getting injured. The percentage of missing values was too low to significantly affect the results. No observation was completely omitted from the analyses.

### Ethical consideration

The study protocol was approved by the institutional review board of the Biomedical Research Foundation, Bangladesh (Ref. no: BRF/ERB/2020/002). Each participant provided informed written consent. However, some illiterate respondents could not sign their names. In these instances, the questionnaire was marked as a verbal agreement.

## Results

### Respondents and metal workshop characteristics

All the metal workers (*n* = 205) who participated were male in this study. The mean age of the participants was 34.89 (SD 10.57) years. Almost two-thirds (62.7%) of respondents attended up to primary education and 27.5% had secondary education. About three-fourths (72.9%) of respondents were the owner while 22.7% of workers were paid. Welding and metal cutting were the most common operations (90.2 and 83.4% respectively) among the respondents. The number of workers was between 1 and 3 in more than half (59.5%) of the workshops. The mean age of the factories was 13 (SD 11.63) years (Table 1).

**Table 1:** Sociodemographic characteristics of study participants

Variables	n	%
***Age***		
Mean (SD)	34.89 (10.57), *n* = 203	
***Highest Education***		
No Education	4	1.99
Primary	126	62.69
Secondary	55	27.36
Higher Secondary	16	7.96
***Occupation***		
Owner	148	72.91
Paid (permanent)	46	22.66
Day Labourer	3	1.48
Trainee	6	2.96
***Marital Status***		
Married	180	88.67
Unmarried	23	11.33
***Monthly Family Income***		
< 10,000	39	19.02
10,000-14,999	67	32.68
15,000-19,999	56	27.32
> =20,000	43	20.98
***Type of Metalwork***^***a***^		
Metal cutting	171	83.41
Welding	185	90.24
Machining	47	22.93
Auto Servicing	22	10.73
Other	22	10.73
***Number of workers in factory***		
Median (IQR)	3 (5–2), *n* = 205	
1–3	122	59.51
4–10	73	35.61
> 10	10	4.88
***Age of factory***		
Mean (SD)	13.00 (11.63), *n* = 205	

^a^Percentages do not add up to 100% because the respondent can select multiple answers

### Injury profile

Out of 205 metal workers interviewed, 170 had a history of injury prior to 1 year of the survey, leaving the annual rate of work-related injury to be 82.9% (95% CI = 77.1–87.8%). Among injured workers, 76.5% experienced injuries more than once in the given period. Lacerations were the leading type of injury (78.2%) followed by musculoskeletal disorders (61.8%) and eye problems (58.2%). The median of lost working days due to injury was 20 days, where the majority (81.1%) of injured workers lost more than three working days. Most (80.6%) of the injury cases were managed by visiting doctors while only 20.6% were managed in the workplace using first aid services. More than two-thirds (68.3%) of workers suffered financially because of severe work-related injuries (Table 2).

**Table 2:** Workers’ history of injury

Variables	n	%
***Injury in the last 1 year***		
Yes	170	82.93
No	35	17.07
***Number of occurrences (n = 170)***		
Once	40	23.53
More than once	130	76.47
***Lost working days (n = 169)***		
Median (IQR)	20 (30–5)	
*< =3*	32	18.93
*> 3*	137	81.07
***Type of Injury (n = 170)***^***a***^		
Lacerations	133	78.24
Skin allergy	16	9.41
Eye problem	99	58.24
Musculoskeletal Disorders	105	61.76
Muscle numbness	33	19.41
Back pain	44	49.41
Shoulder pain	48	28.24
Knee pain	56	32.94
Others	22	12.94
***Immediate measure (n = 170)***^***a***^		
First Aid	35	20.59
Visit Doctors	137	80.59
Visit Hospital	58	34.12
***Suffered financially after injury (n = 205)***		
Yes	140	68.29
No	59	28.78
Don’t know	6	2.93

^a^Percentages do not add up to 100% because the respondent can select multiple answers

### Perception and knowledge

Nearly half of the workers perceived that their working environment was safe and healthy. Regarding OHS-related knowledge, most respondents (99%) were aware of the harmful effect of metal dust (99%), the sound of metal cutting (92.7%), and radiation (89.3%). Almost all (99%) participants recognized the need of wearing safety glasses or goggles, shoes, and masks to protect their health at the workplace (Table 3).

**Table 3:** Knowledge and practice of OHS among workers

Statement	Yes n (%)	No n (%)	Don’t Know n (%)
***Knowledge***			
Exposure to metal dust causes health problems	203 (99.02)	0 (0.00)	2 (0.98)
The sound created during cutting metal causes health problems	190 (92.68)	4 (1.95)	11 (5.37)
Radiation during welding causes health problems	183 (89.27)	1 (0.49)	21 (10.24)
Wearing safety glasses or goggles, shoes, and masks can protect the health	203 (99.02)	1 (0.49)	1 (0.49)
***Practice***			
Do you use PPE?	158 (77.07)	47 (22.93)	0 (0.00)
Do you have a First Aid box in your shop?	19 (9.27)	186 (90.73)	0 (0.00)
Do you need to comply governmental rules and regulations?	13 (6.37)	140 (68.63)	51 (25.00)
Do you have any documented policy/procedure for operations?	3 (1.49)	197 (97.52)	2 (0.99)
Do you have any awareness programs?	6 (2.93)	199 (97.07)	0 (0.00)
Do you conduct risk assessments for your critical process?	63 (30.73)	142 (69.27)	0 (0.00)
Do you have any safety training programs for employees?	4 (1.95)	201 (98.05)	0 (0.00)
Is manual handling very common in your operation?	128 (63.05)	75 (36.95)	0 (0.00)
Do you have any equipment/machines to move objects?	40 (19.51)	165 (80.49)	0 (0.00)
*Type of PPE used (n = 205)*	*n (%)*		
Eye goggles	156 (76.10)		
Gloves	17 (8.29)		
Safety boots	5 (2.44)		
Helmet	6 (2.93)		
Mask	38 (18.54)		
***Self-reported OHS status of workplace***			
Statement: My working environment is safe and healthy	n (%)		
Strongly Agree	42 (20.59)		
Agree	60 (29.41)		
Neutral	37 (18.14)		
Disagree	52 (25.49)		
Strongly disagree	13 (6.37)		

### Safety practices

A large proportion (77.1%) of respondents wore some PPE but the majority (90.7%) of the workshops did not have a first aid box. Almost all workshops had not any documented policy and/or procedure for operations. Only 2 and 2.9% of respondent workers underwent safety training and awareness programs respectively. Two-thirds (68.6%) of respondents thought that there was no obligation to comply with specific guidelines and legislation formulated by the government. No risk assessments were conducted for critical processes in 69.3% of shops. Metal handling was very common in 63.1% of workshops while 80.5% of workers did not have any equipment or machines to move objects. Regarding the use of PPE, eye goggles were mentioned by the majority (76.1%) of the workers followed by masks (18.5%). Safety boots were mentioned by the least (2.4%) (Table 3).

### Factors associated with practice score and injuries

In univariate analyses, both education and monthly family income were significantly associated with the practice score while no significant associations were found for other variables such as age, occupation, marital status, knowledge score, number of workers in a factory, and age of factory. However, the mean practice score among all workers was very low, 1.86 (SD 1.17). Further analysis using multivariate regression showed that education, monthly family income, and occupation were significantly associated with the practice score (Table 4).

**Table 4:** Association between the Practice Score, Sociodemographic Characteristics and Knowledge

Variables (n)	Univariate Analysis		Multivariate Analysis (1)		Multivariate Analysis (2)	
	Beta	*P*-value	Beta	*P*-value	Beta	*P*-value
Intercept			1.84	< 0.001***	2.09	< 0.001***
**Age** (0 = “< 25”, 1 = “> = 25”)	0.09	0.721			−0.03	0.894
**Education** (0 = No/ Primary, 1 = Above Primary)	0.53	0.002***	0.56	0.001***	0.62	< 0.001***
**Occupation** (0 = Owner, 1 = Other)	−0.29	0.104			−0.42	0.056*
**Marital Status** (0 = Unmarried, 1 = Married)	0.08	0.747			0.19	0.513
**Monthly Family Income** (0 = “< 15,000”, 1 = “> = 15,000”)	−0.33	0.041**	−0.36	0.028**	−0.40	0.020**
**Number of workers in factory** (0 = “<=3”, 1 = “> 3”)	− 0.09	0.604			− 0.09	0.607
**Age of factory** (0 = “< 10”, 1 = “> = 10”)	0 .12	0.461			0.09	0.590
**Knowledge Score** (0 = “< 4”, 1 = “4”)	−0.25	0.312			−0.36	0.162

*** *p* < 0.01, ** *p* < 0.05, * *p* < 0.1

The number of workers in the factory and the age of the factory were significantly associated with the occurrence of occupational injury in the univariate models. Only the number of workers was statistically significant in the final multivariate model (Table 5). Workers working in factories with more than three workers were 3.33 times more likely to be injured [AOR = 3.33, 95% CI = 1.16, 9.58] compared to the workers in factories with one to three workers.

**Table 5:** Determinants of Occupational Injury

Predictor Variables	Occupational injury		OR (95% CI)	AOR (95% CI)
	Yes	No		
Age				
Less than 25	21	6	1.00	1.00
25 and above	147	29	1.45 (0.54, 3.90)	1.44 (0.42, 4.91)
Education				
No/ Primary	104	26	1.00	1.00
Above Primary	62	9	1.72 (0.76, 3.91)	1.44 (0.58, 3.58)
Occupation				
Owner	121	27	1.00	1.00
Other	47	8	1.31 (0.56, 3.09)	1.23 (0.34, 4.40)
Marital status				
Unmarried	18	5	1.00	1.00
Married	150	30	1.39 (0.48, 4.03)	1.65 (0.40, 6.79)
Monthly family income				
< 15,000	85	21	1.00	1.00
> =15,000	85	14	1.50 (0.72, 3.14)	1.30 (0.55, 3.08)
Number of workers				
1–3	93	29	1.00	1.00
More than 3	77	6	4.00 (1.58, 10.14)***	3.33 (1.16, 9.58)**
Age of factory				
Less than 10	71	20	1.00	1.00
10 and above	99	15	1.86 (0.89, 3.88)*	1.41 (0.62, 3.19)
Workplace is safe				
Strongly Agree/ Agree	83	19	1.02 (0.39, 2.67)	0.94 (0.31, 2.83)
Neutral	30	7	1.00	1.00
Strongly Disagree/ Disagree	56	9	1.45 (0.49, 4.29)	1.45 (0.43, 4.91)
Knowledge score				
< 4	20	5	1.00	1.00
4	150	30	1.25 (0.44, 3.59)	2.56 (0.76, 8.63)
Practice score			1.21 (0.87, 1.68)	1.27 (0.86, 1.88)

The result of the Hosmer and Lemshow test was > 0.333

Mean VIF = 4.35

*** *p* < 0.01, ** *p* < 0.05, * *p* < 0.1

## Discussion

In this community-based study, the first of its kind in Bangladesh, the majority of small metal workshop workers had experienced at least one injury in a one-year timeframe. We found that laceration, musculoskeletal disorders (MSD) and eye problems were among the most cited injuries. Our study has also revealed the significant gaps in OHS-related knowledge, awareness, and safety practices among metal workers in Bangladesh.

### High annual rate of injuries and their causes and consequences

This study found a very high annual rate (~ 83%) of occupational injuries among metal workers. This finding is similar to other studies conducted in Bangladesh (prevalence of musculoskeletal symptoms among metal workers during 1 year preceding the survey is 85%), India (prevalence of occupational morbidity is 60% among iron/steel workers and annual injury rate is 100% among welders), and Uganda (prevalence of occupational injuries among welders is ~ 88% within 1 year prior to the study) [12, 15, 18, 19]. As compared to construction workers, the rate of injury is higher among workers in our study [20]. Lacerations, MSDs, and eye problems were among the most cited injuries in this study.

The incidence of injuries was not associated with sociodemographic factors including age, education, occupation, marital status, income, or knowledge score in our study. In the case of other occupational injuries (among construction workers), age and education were found to be significantly associated with the history of injury in Bangladesh [20]. As most of the workers in our study were the owner of the factories, the age of the factory was possibly equivalent to working experience or service year. In our study, workers with 10 years or above of working experience were more likely to encounter injuries during 1 year preceding the study. This is in line with the findings from the construction workers of Ethiopia- the likelihood of injury occurrences among those who worked for more than 5 years was higher than among those who worked for a few years [21]. We found that the risk of injury was higher in factories with more than three workers, suggesting that it might be difficult to maintain occupational safety in large factories as there is a lack of proper safety practices and monitoring by the local authority. A similar finding is reported from iron, steel and metal manufacturing industries in Ethiopia where occupational injuries were more common in medium or large size industries than the small size industries [22].

As a consequence of widespread injuries irrespective of sociodemographic factors, a large proportion (~ 70%) of metal shop workers lost several days (median 20 days) of work and suffered financially. Financial suffering can affect workers in the lower-income category the most. The nonexistence of occupational health insurance in Bangladesh could make the low-paid victim families vulnerable to bankruptcy.

### Basic knowledge of OHS is not reflected in practice

We found that almost all workers had correct basic knowledge about OHS and a large proportion of workers reported that their workplaces were safe and healthy. However, the average practice score was not satisfactory among respondent workers. In other words, awareness did not lead to actions. This is evident by the fact that most workshops did not have training programs, safety guidelines, first aid boxes, and regular risk assessment programs. As expected, a higher level of education was associated with a higher practice score since education can of course raise awareness. Interestingly, higher family income was associated with a lower practice score might be because higher incomes are received by highly experienced workers who are confident in avoiding injuries. An in-depth qualitative study is warranted to deeply understand the gap between the knowledge and practice among metal workers in Bangladesh.

We documented that the owners of the shops had higher practice scores as compared to the other employees. This could be comparable to some extent to a study conducted among particleboard workers in Ethiopia where permanent workers had higher knowledge scores as compared to temporary workers. Consequently, permanent workers had better workplace practices [16].

### Unsatisfactory PPE practices

Even though a higher proportion (~ 76%) of workers reported using eye goggles, more than half of them suffered from eye problems. The reason for eye goggles not being able to protect the eyes could be the low quality of eye goggles or using the alternatives of eye goggles such as sunglasses without differentiating among them. For instance, a high proportion of welders in Nepal were found to use sunglasses (not recommended PPE) as these are cheap and comfortable [23]. Most workshops (80.49%) in our study did not have machines to move objects and therefore manual handling could be linked with a higher incidence of MSD in our study.

Specific information on the type of PPE (such as sandals or closed-toe shoes or work boots, clear or polarized glasses or goggles or face shields, and cloth masks or industrial masks) would have been more informative. This was beyond our survey scope because we wanted to keep the questionnaire as simple as possible for the workers with a low level of education. However, from the researchers’ experience at the field level, masks were typically loose-fitting cloth masks, eye wears were mostly clear goggles or face shields. Workers typically wore sandals instead of any shoes let alone safety boots [24]. Given the socioeconomic context of the country, wearing industry-grade masks and any sort of hearing protection are uncommon in the metal workshops at the community level in Bangladesh.

### Non-existence of safety training programs and monitoring by the local authority

We found that almost all respondents (nearly 70% of metal shop workers) were unaware of OHS-related governmental rules and regulations. Most shops did not have written OHS policies or procedures. Importantly, programs for safety training, risk assessment, and awareness were nonexistent in most shops that participated in this study. Proper institutional policy and practice, such as the use of PPE and having institutional training, are associated with a lower risk of injuries, according to studies in India and Iran [15, 25]. The scenario is different in developed countries like Australia where the majority (87%) of employers in the manufacturing sector reported that they provided some health and safety training for each worker in the past 12 months [26].

### Lack of implementation of existing national policy

The Government of Bangladesh adopted the “National Occupational Health & Safety (OHS) Policy 2013”, the first of its kind in the country [27]. The policy covers all industries in the formal-informal sector of Bangladesh including factories, establishments, trade and commerce, the agriculture sector, and all other workplaces to develop the condition of OHS everywhere. Overall, the policy aims to reduce the deaths, injuries, and diseases due to workplace hazards so that the workers’ productivity increases. The obligations of the policy include to pre-inform all workplace employees about potential accidents, health and safety risks, providing basic training for workers, using appropriate technology, and infrastructural development to ensure safety. However, our study findings indicate that the implementation of the National OHS Policy 2013 is not widespread. Owners’ associations are responsible to ensure medical care and compensation for accidents and rehabilitation of injured workers, according to the policy. Employers are also obliged to formulate their own specific OHS policy for their workplace and follow that. These are not practised in the informal, unregistered, and community-based participant workshops of our study as a consequence of negligible monitoring of social and workplace compliance. Insurance policies and social safety nets are not present to compensate for occupational health hazards although the National Plan of Action on OHS 2021–30 of Bangladesh includes the introduction of an employment injury insurance scheme by 2026.

The OHS practices in developed and western countries, for example in Australia, show a positive scenario on the other hand. There are some factors that contribute to OHS practices and performances in developed countries including legislative and regulatory framework, innovative OHS initiatives, increased awareness and Government support. All businesses in Australia must comply Work Health and Safety Act 2011 and Work Health and Safety Regulations 2011, whereas every business must have a policy for managing OHS. SME owners and managers recognise the penalties they face for failure to maintain healthy and safe working environments in Australia. They are also aware of the potential costs of death and injury to both their employees and their organisations. In large businesses, formal OHS management teams are engaged to help provide the necessary compliance with regulations to ensure the health and safety of the workforce [28].

### Policy implications of our findings

Bangladesh has formulated its first OHS profile in 2015 presenting the OHS-related policy landscape and regulatory frames; OHS-related institutions and collaborative mechanisms; state of OHS inspection; and occupational diseases, hazards, and risks for workers. The OHS profile 2015 reemphasizes the use of PPE, the necessity of training, and the formulation of a health and safety committee. Although the informal sector accommodates a large number (85%) of employees in Bangladesh [5], there are no major attempts to improve workplace safety in the informal sector. Our findings can contribute as a supplementary document to the National OHS profile by providing important facts on the informal metal sector. Proper workplace policy is needed to ensure good workplace practice including workers’ safety training, risk assessments, documentation, and availability of first aid, PPE and necessary equipment. Safety net coverage can be introduced to financially harmed and injured workers. Some of these protocols can only be seen in Readymade Garment (RMG) sectors as per international stakeholders’ demand. A complete framework needs to be developed focusing on small industries and workshops to minimize occupational health hazards and ensure social safety nets for the ignored metal workers. Age and size of factories were identified as significant factors associated with occupational injury. Therefore, necessary provisions in the OHS policy are needed to maintain safety at large and old factories. A cost-benefit analysis in the shipbuilding industry of Bangladesh showed that investments in the workplace and environmental safety (new clinic, PPE, training) decrease injuries and increase efficiency [29]. Such analysis should be done in the metal industry too in the near future. The National Plan of Action on OHS 2021–30 suggested OHS coverage to SMEs and informal economy workplaces, in which we are still lagging. Furthermore, in the context of Bangladesh, some simple and inexpensive ways should be promoted to improve workers’ health and safety as well as the health and wellness of their families. These include proper housekeeping, sanitation, and hand washing.

### Limitations of the study

This study has a few limitations. First, cross-sectional study design and recall bias are the main limitations. Second, our study findings may not be generalizable to all community settings in Bangladesh. However, the socio-demographic factors of this study area are similar to the other 33 district towns in Bangladesh [13]. Third, as most of the respondents are the owner and paid permanent employees, it might be difficult to generalize the results for temporary or vulnerable employees. This is important because they are generally less skilled or less trained having low knowledge scores [16], which means that they are more likely to get injured in the workplace. Fourth, the knowledge-related questions only covered very fundamental issues. Knowing the attitude of the workers besides knowledge and practice is also important to formulate policies but the attitude of the workers is not covered in this study. Fifth, we fully acknowledge that, by nature, there is no classic referent group for comparison, as might be possible with resources for a larger study design.

## Conclusion

To the best of our knowledge, we, for the first time in Bangladesh, depicted the overall health and safety practices among metal workers in a community setting as this study covered most metal workshops in the study area where the majority of the owners participated. The annual rate of occupational injury was high among metal workers in the study area because of the absence of implementation of OHS policies and monitoring by the relevant local authority. It is expected that these baseline findings would contribute to the designing of interventional studies and effective policy formulation in Bangladesh.

Awareness campaigns should be launched targeting the metal sector. Common mass media like television, social networks etc. should be used for this campaign. OHS skills development should be integrated effectively into relevant education and training programs. OHS awareness programs for the owners of these shops should be organized and enforced as part of the registration of their businesses. Training should be provided to the metal workers and those providing OHS education, training, and advice need to have appropriate capabilities. Government should support the informal metal working sector to increase awareness and skills for the prevention and proper management of injuries and risks, and to ensure access to safety equipment and a safe environment.

## Data Availability

The datasets used and/or analyzed during the current study are available from the corresponding author upon reasonable request.

## Abbreviations

GDP: Gross Domestic Product
ILO: International Labor Organization
OHS: Occupational Health and Safety
DIFE: Department of Inspection for Factories and Establishments
LFS: Labour Force Survey
REDCap: Research Electronic Data Capture
PPE: Personal Protective Equipment
MoLE: Ministry of Labour and Employment
SME: Small and Medium-sized Enterprises
RMG: Ready-made garment
